# 

**Published:** 1886-02

**Authors:** 


### INDEX.

A

A compliment 695
A druggist's mistake 183
Acne pustula 606
Advice to young men 66
A manual of Botany 163
A plea for purity 677
Alcohol, physiological therapeutical, properties of 455
American Medical Association 220
American Surgical Association 188
An act to establish a State Board of Medical Examiners 362
An imposter 180
Animal food, is it essential 539
Announcement for 1886, 690
Aphasia 385
Asylum tragedies 373

B

Books and pamphlets received 166, 358, 435, 566
Book reviews: A Hand-book of Medical Sciences 629, Acne, its Etiology, Pathology and Treatment 632, A Hand book Therapeutics 560, A System of Practical Medicine 37, 295, 633, A Practical Treatise on Nasal Catarrh 562, A Treatise on Nervous Diseases 565, A Practical Treatise on Diseases of Children 165, 566, A System of Obstetric Medicine and Surgery 682, A Treatise on Diseases of the Ear 49, A Manual of Diseases of Women 634, An Atlas of Clinical Microscopy 685, An Epitome of Diseases of Eye, Ear, Throat and Nose 303, Bill Arp's Scrap Book 48, Cholera 433, Diseases of the Skin 564, Diseases of the Nervous System, especially Women 163, Diseases of Urinary and Male Sexual Organs 241, Epilepsy and other Convulsive Diseases 635, Fawns Manual of Chemistry 686, Hand-book of Diseases of the Skin 304, Hand-book of Physiology 302, Hay Fever 354, Index Catalogue, Surgeon-general's office vol. 6, 506, Inebriism 563, Insomnia and other Disorders of Sleep 564 Key to Smith's Diagram of Parliamentary practice 687, Minor Surigcal Gynecology 238, Milk Analysis and Infant Feeding 687, Modern Medical Therapeutics 47, Pharmacology, Theraputics and Materia Medica 681, Perils of American Women 357, Poisons, their Effects and Detection 355, 684, Renal and Urinary Affections 686, The first Three Years of Childhood 686, The Modern Diseases of Children 301, The Management of Labor 565, The Uses of the Microscope 363, The Oleates 358, The Physician Himself 164, The Technology of Bacteria Investigation 434, Therapeutics of the Respiratory Passages 164, Treatment of Nervous Diseases 43,
Transactions New York Medical Association 303, Transactions Texas Medical Association 683, Transactions Vermont Medical Society 300, Urinary and Renal derangements 356, Waisting diseases of infants and children 354, Young folks' Physiology 46.
Brain abscess of 284, Thrombosis and embolism 609.
British Medical Association 441, and homœopaths 443
Bubo virulent 672
Burns 139, Treatment of 145.

C

Cæsarean section 465
Cancer of the Tongue 171
Can't be trusted 315
Cellulitis Peri-uterine 416
Cephalæmatoma 470
Chancroid 100
Chloroform, death from 181
Chlorosis 271
Cholecystotomy 378
Cholera Asiatic 368, Vaccinations against 232
Cocaine Muriate 18, 34, 658, cost of 441, habit of 676
Colleges: Atlanta Medical 120, Southern Medical 122
Constipation 384
Correspondence: 34, 289, 426, 480, 550 624
Craniotomy, Papal and Talmudical opinions of 689
Crowner's quest law 115

D

Diptheria, a new method of treating 449
Dirt Eater, post mortem examination of 351
Dislocation of the elbow 641
Disseminating Knowledge and doing good "a la mode" 691
Duodeno-Cholecystostomy 395, 588
Dysentery 315

E

Electricity as a stimulant in Cardsac an respiratory failure 89, in Aconite poison 90, In Choloraform poisoning 90, In Opium poison 91
Empyema 675
Endo-Arteritis, chronic 252
Epididymitis 96
Epilepsy 377
Epistaxis, treatment of 384
Epithelioma 544, Of the clitoris 669
Explosive physic 575
Eye ball, enucleation of 32, Eviceration of 673

F

Faith cures 636
Fecundily, Extraordinary 767,
Fever : Catarrhal 8, Hay, cocaine in 604, Typhoid 133. Yellow, inoculations against 640, Government commission for investigating 763
Fistula in ano, treatment of 333
Fracture, dressing 235, plaster paris 268, 331

G

Georgia Press Association 124
Glaucoma 348, Result of injudicious use of atropia 341, Treatment of 350

H

Hematuria, malarial 382, 412
Heart beats 280
Hemorrhage post-partem 27, Vaginal following coitus 294
Hemorrhoids, treatment of by injections 375
He never made a mistake 696
Hernia femoral 20, Inguinal 26
Herpes Zoster 666, Treatment of 667
Hydrocele 330
Hydramnios 404
Hydrophobia, deaths from 694, Pasteur's work in 702, Precautions against 695
Hyperidrosis 391, Salicylic acid, suet in 442

I

Illinois State Board of Health 188, 142
Importance of early treatment of the Insane 502
In bad taste 510
Infants, dressing of new born 159
International Medical Congress 360, 444 511, 574, Georgians officers in 376, And the medical profession of Boston 378, 383, Of Philadelphia 380, Of Baltimore 381, Of Washington 383, Muddle 439, Fiction *vs.* facts 679
Irido-chrooiditis following meningitis 233, Treatment of 234
Items : 59, 128, 179, 251, 314, 372, 441, 507, 572, 638, 694

L

Labor, a case of prolaspus of vagina preceding 271
Lactation arrested and deficient 75 In a woman sixty years old 133
Laparotomy for intestinal obstruction 407 For gun shot injury 444
Lipomata of the obit 473
Locating the association 426, 480

M

Mastitis 13
Maternal impressions 186
Meanest man on record 510
Medical Association of Georgia. 123, 177 Meeting of 146, Report of committees on publication 148, New members 149, Prize essays 150, Sections 151, Voluntary contribution 151, Auditing 154, Nominating 155, Locating the association 157 treasurer's report 254, President's address 193, Orator's address 257, Shall it be located 242, Medical legislation needed 312, Roll of members, 782, In memoriam 789.
Medical Society of Tennessee, officers of 180
Medical Society of Alabama 185
Menstruation in an infant 317
Meteorological summary 63, 185, 255, 380 442 511, 576, 643, 707.
Micophone in diagnosis 129, 291
Missed him 372
Monstrosities 474, 592, 700

N

Necrosis, result of gun-shot wound 329.
Neuralgia, facial muriate of ammonia in 373
Night calls, how to avoid them 383
Night Sweats, ergot in 374.
Nursing and the training of 259

O

Obituaries : Dr. E. S. Gaillard 58, Dr. R. F. Wright 62, Dr. J. H. Logan 175 Dr. Oliver S. Taylor 252, Dr. W. D. Mathews 441, Dr. D. B. Searcy 442, Dr. S. H. Gray 504, Dr. F. A. Stanford 507, Dr. R Campbell 508, Dr. Richard Mc-Sherry 572, Dr. Jno. L. Atlee, Sr. 572, Dr. W. H. Coggeshall 576, Dr. O. R. Ford 638, Dr W. B. Carpenter 642, Dr. E. Fitzgerald 699, Mrs. Dr. Howell 640, Dr. Joseph A. Eve 762, Dr. L. A. Dugas 768.
Ophthalmia, catarrhal, cocaine in 135
Opium poison, nux vomica in 535
Orchitis 96 Phytolacca decandra in 340
Osteo-arthritis of knee joint 286
Our Advertisers 63, 191, 256, 320, 448, 512, 644, 707

Our Journal, vol. II 50
Our Portrait Gallery: Dr. J. Mc F. Gasten 52, Dr. T. O. Powell 110, Dr. J. W. Bailey 173 Dr. Tomlinson Fort 243, Dr. Richard Banks 305
Ovariotomy, conditions of success in 1, Spray in 36, In Battey Infirmary 437, Conservitive 623

P

Paralysis result of fall 674
Personals: Dr. W. P. Harden 59, Dr. Henry Wile 59, 372 Dr. J. H. Logan 59, Dr. D. W. Hammond 59, Dr. A. W Calhoun 59, 509, Dr. R. C Eve 59, Dr. J. F. Lancaster 60, Dr. Robert Battey 61, Dr. R. O. Cotter 61, Dr. N. O. Harris 128, T. O. Powell 179 Dr. E. H. Richardson 179, Dr. J. B. Roberts 180, Dr. J. W. Hollinsworth 180, Dr. D. B. Searcy 182, Dr J. P. Taylor 189, Dr. F. M. Brantly 189, Dr. R. H. Taylor 189, Dr. F. W. McRae 251, Dr J. S Macon 251, Dr. A. C Clements 251, Dr. J. D. Wilson 251, Dr. W. A. Crow 255, Dr. C. F. Benson 314, Dr. R. J. Nunn 314, Dr. C. D. Smith 314, Dr. J. C. Le Hardy, 372, Dr. M. A. Starr 372, Dr. J. M. Head 507, Dr. M. H. O'Daniel 507, Dr. Harry Huzza 508, Dr. J. E. Cook 572, Dr. H. F. Campbell 572, Dr. W. F. Holt 572, Dr. C. W. Nutting 574, Dr. A. W. Wilder 639, Dr. J D. Pendergrass 639, Dr. Jno. M. Johnson 639, Dr Eugene Foster 639, Dr. A. H. Shi 639, Dr. J. W. Clements 639, Dr. A. G. Hobbs 640, Dr. Jos. A. Eve 640, Dr A. P. Thornton 694, Dr. E. N. Shaw 694, Dr. M. Salm 694.
Perineum, lacerations of 87
Pessaries, the use and abuse of 577
Phimosis, death after operation for 700,
Polypus of the external auditory canal 137, Treatment of 138
Premature death, the extent, cause and prevention of 194
Publisher's announcement 178

Q

Quinine, cheapness of 187

Quacks 250

R

Rattlesnake bite 338, Permanganate Potash in 339
Remarkable longevity 375
Rheumatism 229
Rupture of the small intestines and bladder 344

S

Sex, how to predict it 181
Shall our young men study the classics 645
Small pox contracted by correspondence 253
Society Reports: Atlanta Medical and Surgical Union 20, Atlanta Society of Medicine 422, 476, 668, Baltimore Academy of Medicine 673, Macon Medical Society 13, 96, 159, 229, 539, 609, Medical Association of Georgia 146, New York Acadamy of Medicine 89, 695
Stomach, spontaneous rupture of 549
Syphilis 100, Hereditary 655, Importance of thorough diagnosis and long continued treatment 513, Specific bacili of 374, Of the urethra 668

T

Tabes Mesenterica 278
The deadly tea-pot 184
The Journal 252, 254, 291, 314, 317
The Lex Coronaria 321
The Local Option Act 569
The Local Option Bill and the Medical Profession 551
The man with interesting cases 702
Tongue deformity of 184
Tonsils, Hypertrophy of 281
Tracheotomy 603
Trachomatous growths upon the vocal cords 98
Trichinosis 698, Death from 441
Tumors: fibro-cystic 29 Of the meatus urinarious 475, uterine treatment of 81, Ovarian treatment of 81
Two frauds 574

U

Ulcers: of the cornea 618, Varicose 547
Umbilical Cord, dry dressing for 35
Uterus: displacements of 611, Double, a case of 275, fibroid tumors of 476, Lacerated cervix of, Emmett's operation in 335, 675, Polypus of 664, Senile atrophy of 422

V

Vaginismus, cocaine in, 509
Verruca vulgaris 479

W

Water, impure, danger of 250, mineral 705
Water gas, dangers of 112
Wells and bacteria 501
What is dental surgery 237
What shall the harvest be 186

